# Design and Performance Analysis of a Multilayer Sea Ice Temperature Sensor Used in Polar Region

**DOI:** 10.3390/s18124467

**Published:** 2018-12-17

**Authors:** Guangyu Zuo, Yinke Dou, Xiaomin Chang, Yan Chen, Chunyan Ma

**Affiliations:** 1 College of Electrical and Power Engineering, Taiyuan University of Technology, Taiyuan 030024, China; zuoguangyu0030@link.tyut.edu.cn (G.Z.); chenyanlxq@163.com (Y.C.); tyutchyma@sina.com (C.M.); 2 SOA Key Laboratory for Polar Science, Polar Research Institute of China, Shanghai 200136, China; 3 College of Water Resources Science and Engineering, Taiyuan University of Technology, Taiyuan 030024, China; changxiaomin@tyut.edu.cn

**Keywords:** multilayer sea ice temperature, low temperature, design, performance analysis

## Abstract

Temperature profiles of sea ice have been recorded more than a few decades. However, few high-precision temperature sensors can complete the observation of temperature profile of sea ice, especially in extreme environments. At present, the most widely used sea ice observation instruments can reach an accuracy of sea ice temperature measurement of 0.1 °C. In this study, a multilayer sea ice temperature sensor is developed with temperature measurement accuracy from −0.0047 °C to 0.0059 °C. The sensor system composition, structure of the thermistor string, and work mode are analyzed. The performance of the sensor system is evaluated from −50 °C to 30 °C. The temperature dependence of the constant current source, the amplification circuit, and the analog-to-digital converter (ADC) circuit are comprehensive tested and quantified. A temperature correction algorithm is designed to correct any deviation in the sensor system. A sea-ice thickness discrimination algorithm is proposed in charge of determining the thickness of sea ice automatically. The sensor system was field tested in Wuliangsuhai, Yellow River on 31 January 2018 and the second reservoir of Fen River, Yellow River on 30 January 2018. The integral practicality of this sensor system is identified and examined. The multilayer sea ice temperature sensor will provide good temperature results of sea ice and maintain stable performance in the low ambient temperature.

## 1. Introduction

Arctic sea ice has been a sensitive indicator of climate change and provided the details of the complex atmosphere–ice–ocean interaction, which may affect the global climate and cause extreme weather [1,2,3]. To further improve the fundamental understanding of the mechanism of sea ice rapid changes, accurate representations of temperature profiles of sea ice are urgently demanded. However, most temperature automatic detection equipment used in the conventional environment cannot be directly applied to the observation in the Arctic and Antarctica, due to the extreme cold environment. Conventional temperature sensors include resistances, thermocouples, bimetallic structures, which are metallic, and semiconductors such as diodes and chip integrated circuits [4,5,6]. Metallic temperature sensors placed in sea ice may cause local melting in the sea ice and then liquid water may appear around the sensors affecting the measurement of sea ice temperature. Semiconductor temperature sensors are considered to have a strong nonlinear relationship between temperature and output signals, resulting in rarely use for temperature measurements in the cold area. So far, temperature measurement systems based on optical fiber sensing have been developed but not been used for observation of sea ice in the Arctic Ocean because the vulnerability of fibers does not adapt to the complex sea ice environment. Thus, some polar-specific observing equipment have been developed and deployed. Ice mass balance buoys (IMBs) and sea ice mass balance buoys (SIMBs) are the most widely deployed in the Arctic Ocean to automatically observe sea ice temperature profiles [7,8,9,10]. However, these buoys are only with temperature accuracy of 0.1 °C [11,12], which may unacceptable for ice mass balance studies. The IMB buoy has only 45 temperature points, but three armored cables are exposed to the air, resulting in inconvenient installation and reduced stability. And for the SIMB, discriminating ice thickness based on sea ice temperature profiles is mainly done manually [13,14,15,16], which may bring in errors and cause great uncertainty. The states of the art in sea ice temperature sensor are shown in Table 1.

In these contexts, this paper focused on designing a multilayer sea ice temperature sensor with higher accuracy used in the polar regions. Section 2 describes the sensor system composition, structure of the thermistor string, and work mode of the sensor system. Section 3 gives results of the experiments on low temperature of the temperature measurement accuracy and temperature dependence of the sensor system from −50 °C to 30 °C. In Section 4, a compensation algorithm is proposed based on the results of laboratory experiments to amend the error caused by low temperature. An ice thickness discrimination algorithm theory is designed and proposed in Section 5 to realize the automatic inversion of ice thickness based on the sea ice temperature profiles only. In Section 6, the results of field experiments in Yellow River are examined to evaluating the stability and accuracy of the sensor system in the cold region. The conclusions are present in final section.

## 2. Design and Analysis on the Multilayer Sea Ice Temperature Sensor 

### 2.1. Sensor System Composition 

**Figure 1:**
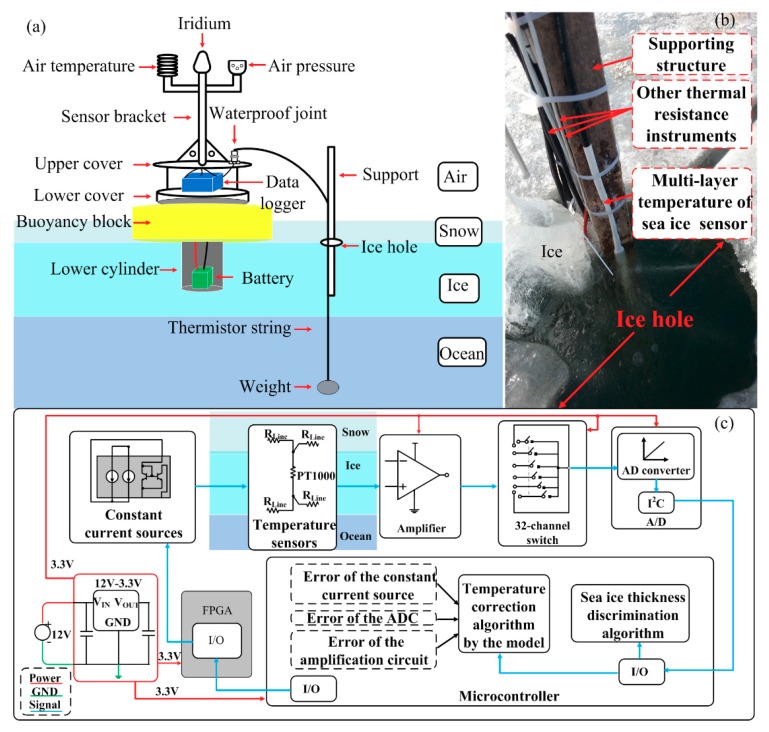
(**a**) System composition of the proposed multilayer sea ice temperature sensor. (**b**) The multilayer sea ice temperature sensor and other thermal resistance instruments installed in Wuliangsuhai, Yellow River in 2018. (**c**) Block diagram of the multilayer sea ice temperature sensor.

The system composition of the proposed multilayer sea ice temperature sensor is shown in Figure 1. The sensor system includes a thermistor string and a buoy. The multilayer sea ice temperature can be measured by the thermistor string, which penetrates air, snow, ice and ocean. The top of the thermistor string is connected to the waterproof joint on the buoy, while the bottom of the thermistor string is connected to the weight to make the thermistor string in a vertical attitude. The thermistor string is installed through a support structure and placed in the ice through an ice hole (Figure 1b). The design of buoy is focused on system portability which means easy deployment and installation, and reducing the impact on sea ice. As shown in Figure 1a, the buoy consists of a main body, a sensor bracket, an electronic cabin as a carrier for the data logger, a buoyancy block and a lower cylinder for placing the battery. An air temperature sensor, an air pressure sensor and an Iridium model used for remote data transmission are fixed on the sensor bracket. The air temperature sensor and air pressure sensor can provide basic environmental data for the observation of sea ice. A waterproof joint is attached to the upper cover and connected to the top of the thermistor string. The data logger is located in the space between the upper and lower covers. This data logger is designed by Taiyuan University of Technology and in charge of obtaining meteorological data (air temperature and air pressure) and sea ice temperature profile data. The correction of measured sea ice temperature profile and calculation of sea ice thickness are also realized by the data logger. In summer, the surface of the sea ice, especially where the automatic observation equipment is installed, will be partially melted to form a melting pond. The formation of the pool may tilt the buoy as the sea ice melts into water. The role of the buoyancy block is to ensure that the buoy stands upright when local melting on ice occurs. When the buoy is installed, the lower cylinder is placed into the drilled ice hole. The lower cylinder acts as a carrier for the battery, bringing the battery closer to the higher temperature seawater to ensure the normal operation of the battery. 

As shown in Figure 1b, the chip-type metallic temperature sensors (PT1000) soldered on the flexible printed circuit boards (FPCBs) are sealed into a white heat-shrink tube to build a thermistor string to avoid direct contact with ice. The length of the thermistor string is 4.5 m, which can meet the measurement range of most sea ice in the Arctic Ocean and Antarctica. The temperature sensor interval is set as 0.03 m. Each 4.5 m-long temperature chain is connected by 15 FPCBs, which are 0.3 m long. The length of the copper line of each PT1000 remains the same when the FPCBs of the thermistor string are formulated. 

The block diagram of the multilayer sea ice temperature sensor is illustrated in Figure 1c. Based on the demands of observation of sea ice temperature, the sensor system should have at least 150 temperature sensitive components to complete temperature measurement at the same time as few cables as possible, if not, it is difficult to avoid damage to exposed cables in extreme environments. In Figure 1c, only one temperature sensitive component (constant current source and temperature sensor) is shown and the implementation of the others is the same as this. The multilayer sea ice temperature sensor system is composed of constant current sources and temperature sensors, amplifiers, 32-channel switches, an ADC circuit, a microcontroller, a field programmable gate array (FPGA) and a power supply circuit. The constant current source circuit (REF200, Texas Instruments, Dallas, TX, USA) generates a constant current of 0.3 mA at a supply of 3.3 V, which provides a stimulus for a PT1000. The amplifier can amplify the analog signal output by the temperature sensor. The analog voltage resolution must be set less than 0.01173 mV to realize the accuracy of temperature measurement better than 0.01 °C. Given that 16-bit AD converter is selected, the amplification circuit (INA118, Texas Instruments, Dallas, TX, USA) is set to realize the voltage of 0.050354 mV. The multisignal channel circuit developed by analog multiplexers ADG732 (Analog Devices Inc, Norwood, MA, USA) has capability of signal isolation, ensuring that multiple signals passing through the multisignal channel circuit without interference and distortion. The inputs of the multisignal channel circuit are connected to the outputs of the amplification circuit. Then one of 32 inputs is switched to a common output connected to the ADC. The ADC circuit is designed by a 16-bit low-noise analog to digital converter (ADS1100, Texas Instruments, Dallas, TX, USA), which is assembled a compatible I^2^C serial interface connected to the microcontroller by one cable. The low-power microcontroller (MSP430F5438A, Texas Instruments, Dallas, TX, USA) is in charge of data processing by inverting sea ice thickness using a sea-ice thickness algorithm and correcting measured temperature using a temperature correction algorithm. The temperature measurement accuracy of the sensor system is improved to better than 0.01 °C. The time response of the sensor system is 200 µs. The FPGA (Cyclone10LP025, Altera, San Jose, CA, USA) is controlled by the microcontroller and can control the switching of the working and sleep modes of the constant current sources and the 32-channel switches. The power supply circuit provides voltage grades for the sensor system. 

### 2.2. Structure of the Thermistor String

**Figure 2:**
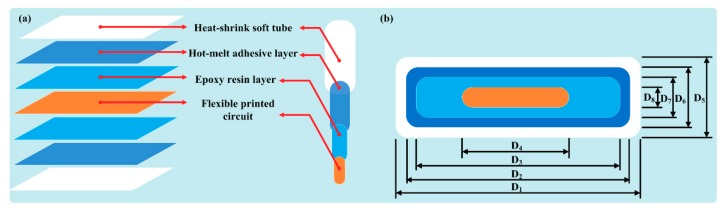
(**a**) Inner structure of the thermistor string. (**b**) Cross-sectional view of the thermistor string.

The inner structure and the cross-sectional view of the thermistor string are schematically shown in Figure 2a,b.

As can be easily seen, the thermistor string is composed of four layers from outside to inside: a heat-shrink soft tube layer, a hot-melt adhesive layer, an expoxy resin layer, and a FPCB. As shown in Figure 2b, the length of the heat-shrink soft tube layer, the hot-melt adhesive layer, the expoxy resin layer, and the FPCB is defined as D_1_, D_2_, D_3_ and D_4_, respectively. And the width of four layers is D_5_, D_6_, D_7_ and D_8_, respectively. The size parameters of the four layers are indicated in Table 2.

### 2.3. Work Mode of the Sensor System

**Figure 3:**
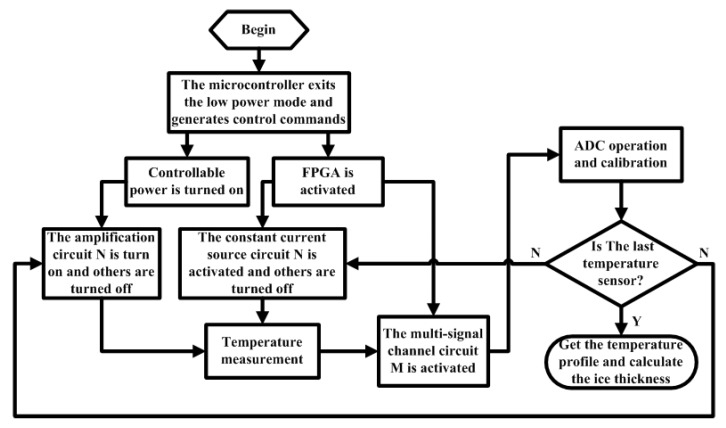
The flow chart of temperature measurement.

Figure 3 shows the flow chart of the multilayer sea ice temperature sensor for temperature measurement. If it is time to collect, the microcontroller will exit the low power mode and enter the work mode. After the sensor system is initialized, the microcontroller send the command to FPGA and will be ready to process data. The FPGA is controlled by the microcontroller to determine which constant current source and amplifier to turn on to realize temperature measurement. If it is not the last sensor of the thermistor string, the temperature measurement process will be repeated until all temperature sensors have completed temperature acquisition. The temperature data will be corrected based on the temperature correction algorithm. Then the sea ice temperature profile and sea ice thickness can be obtained. The label N of the constant current source and the amplifier must match the label M of the multisignal channel switch to ensure the signal is correctly acquired by the ADC circuit.

## 3. Performance Evaluation of the Sensor System

In the polar region, low temperature is the primary challenge for operation of automated equipment. Our multilayer sea ice temperature sensor may affect by its instability and inadaptability at the range of temperatures (−50 to 30 °C). However, we do not fully be aware of the low temperature dependence of the sensor. The potential temperature dependence of the multilayer sea ice temperature sensor needs to be assessed. We focus on the current accuracy and stability of REF200. It is important for the ADC circuit to be evaluated and assessed of the accuracy of AD conversion. The performance of the amplification circuit at low temperature is in evaluation simultaneously.

### 3.1. Temperature Dependence of the Constant Current Source 

**Figure 4:**
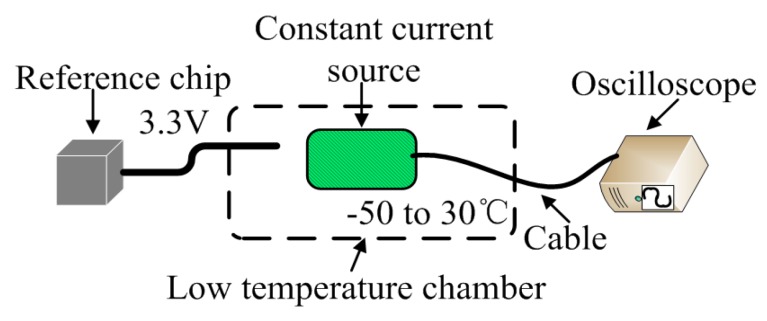
The schematic diagram of the experimental setup of constant current source.

The schematic diagram of the experimental setup of constant current source is shown in Figure 4. In the experiment of constant current source, we put the whole circuit into a GDJS-series high-low temperature test chamber at a stable environmental temperature over a range of −50 °C to 30 °C. The supply voltage of the constant current source was generated by a nominal 3.3 V voltage reference chip. We measured the output of the constant current source circuit by the voltage using a 1KΩ non-temperature drift reference resistor by an oscilloscope (MSO70404C, Tektronix, Beaverton, OR, USA). During the experiment, the interval of measured temperature is set as 5 °C. At each measured temperature, the high-low temperature test chamber maintained the temperature for 20 min in which the measurement repeated 300 times. We took the average values to minimize the statistical error.

**Figure 5:**
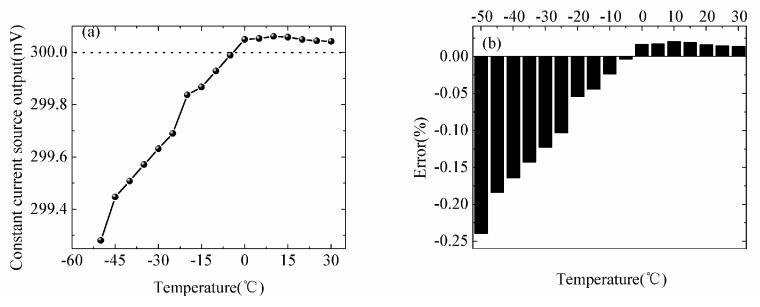
Temperature dependence of (**a**) the output of the constant current source (the measured current values were converted into voltage values), (**b**) error of the output.

The experiment and evaluation results are showed in Figure 5. The results in Figure 5a demonstrate that the temperature dependence of the constant current source is in a narrow range and increase roughly in a parabolic manner. The minimum value of the constant current source is 299.281 mV at −50 °C, which is at the lower limit of the experimental temperature range. The maximum value is 300.061 mV at 10 °C. The average value of the output is 299.83 mV and the standard deviation is 0.249 mV. We calculated coefficient of variation of the output is 0.0831% over the range of experimental temperatures. The errors of the output of the constant current source are shown in Figure 5b. And they indicate that maximum and minimum values are −0.239% at −50 °C and −0.0036% at −5 °C, respectively. Therefore, the experiment results show that the constant current source has excellent stability of low temperature. In the multilayer temperature measurement of sea ice, the biases and influences caused by low-temperature can be effectively reduced by the use of the constant current source.

### 3.2. Temperature Dependence of the ADC Circuit

**Figure 6:**
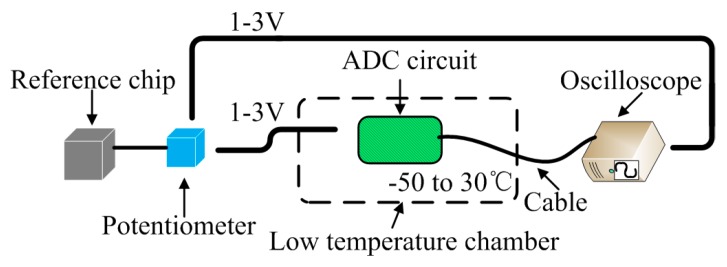
The schematic diagram of the experimental setup of analog-to-digital converter (ADC) circuit.

The schematic diagram of the experimental setup of ADC circuit is shown in Figure 6.

**Figure 7:**
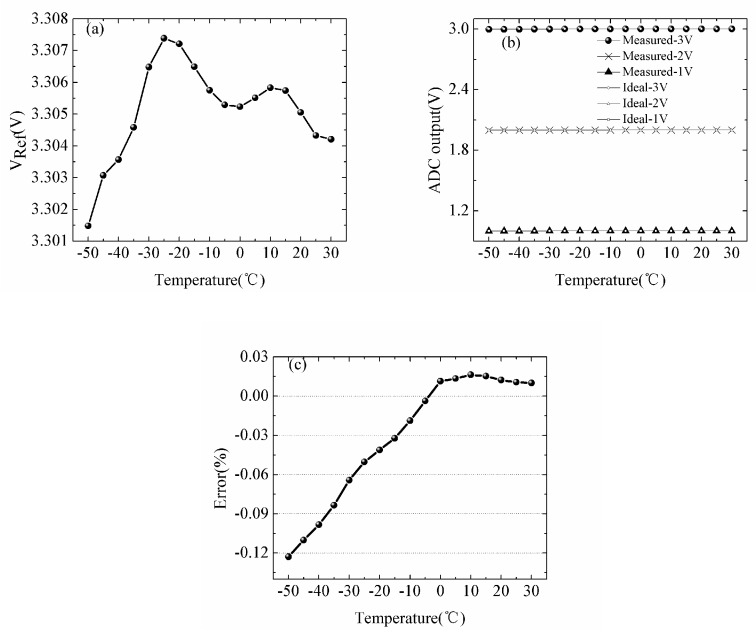
Temperature dependence of (**a**) the reference voltage, (**b**) the output of the ADC circuit, (**c**) the error of the ADC.

Similar to the experiment of the constant current source, we put the ADC circuit into the high-low temperature test chamber with same temperature range. And an auxiliary test circuit is built to complete experiment by providing the adjustable input voltages which simulate the actual detection process. The input voltage generated by a nominal 5 V voltage reference chip and a potentiometer. The range of input is selected from 1 V to 3 V. We measured the output of the ADC circuit by the oscilloscope. At each input, the experiment was cover −50 to 30 °C range. The results in Figure 7a demonstrate that the minimum value of the reference voltage is 3.3015 V at −50 °C, the maximum value is 3.3071 V at −30 °C, the average value of output is 3.30533 V and the standard deviation is 0.001522 V. We calculated coefficient of variation of the reference voltage (0.046%). The results in Figure 7b represent that the measured ADC outputs can be considered stable from our temperature range. The error between the measured output of ADC circuit and the ideal value is shown in Figure 7c. The minimum error of the ADC circuit is −0.00367%. The maximum value is 0.01228% at −50 °C. The results of experiment proved that the temperature-induced error of the ADC circuit is small and acceptable for our design of the sensor system.

### 3.3. Temperature Dependence of the Amplification Circuit

**Figure 8:**
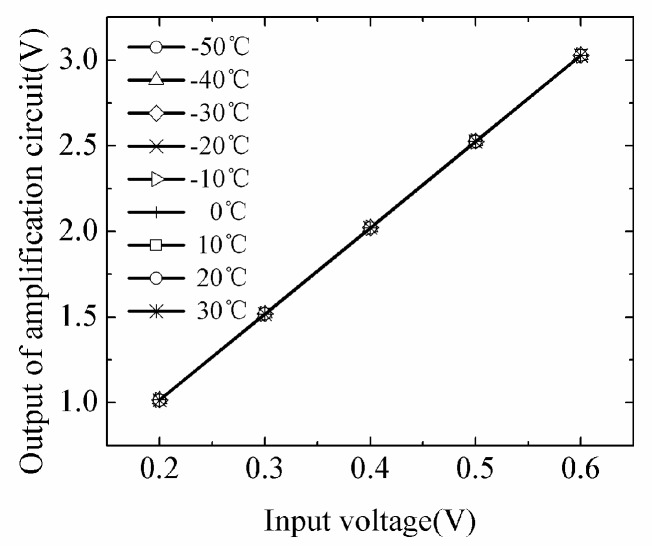
Temperature dependence of the amplification circuit.

The results in Figure 8 demonstrate the temperature dependence of the amplification circuit. 

The adjustable input voltages are generated by the auxiliary test circuit which is used to do the experiment of the ADC circuit. The range of input is selected from 0.2 V to 0.6 V which the interval of input is 0.1 V. We measured the output of the amplification circuit by the oscilloscope. Under the input voltage of 0.2–0.6 V, the output of amplification circuit has strong upward trend proportionately with the increase of the input. For each input, the R^2^ of the amplification circuit output is bigger than 0.999 at each measured temperature.

## 4. Temperature Correction Algorithm for the Sensor System

As mentioned above, the evaluation reveals that low temperature performance of the sensor system maintained a strong stability at temperature range from −50 to 30 °C. However, we still need to develop an approach to correct and minimize the deviation of measuring temperature. The relationship between sea ice temperature, *T_ice_*, and resistance of PT1000 could be approximated as follows.

(1) $$T_{ice}=a(T)R^{2}(T)+b(T)R(T)+c(T)$$

Since *R(T)* could be calculated by the output of the ADC circuit and the corresponding current *I_ref_(T)*, thus, Equation 1 could be described as follows.

(2) $$T_{ice}=a(T){\left[\frac{V_{out}(T)}{I_{ref}(T)}\right]}^{2}+\frac{b(T)V_{out}(T)}{I_{ref}(T)}+c(T)$$

The relationship between sea ice temperature *T_ice_* and resistance of PT1000 could be converted to the relationship between sea ice temperature and the output of ADC *V_out_(T)* by

(3) $$T_{ice}=a(T,I^{2})V_{{}_{out}}^{2}(T)+b(T,I)V_{out}(T)+c(T)$$

**Figure 9:**
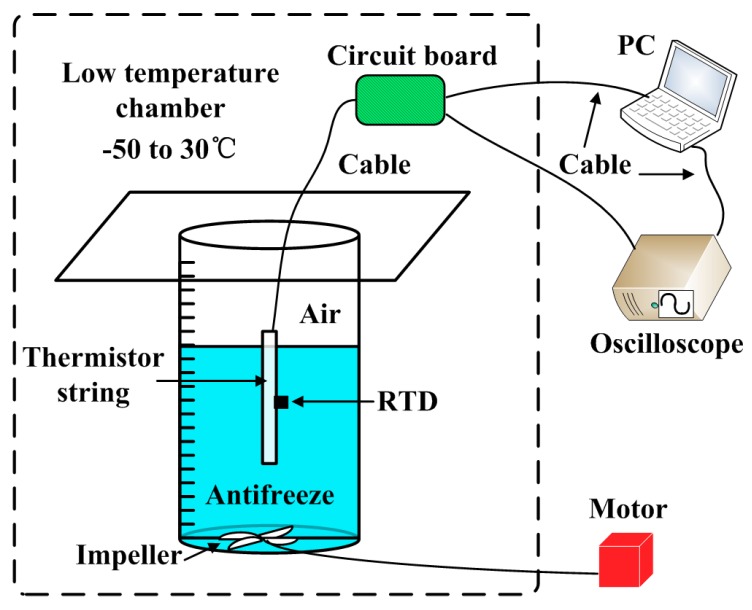
The schematic diagram of the experimental setup of temperature correction.

The schematic diagram of the experimental setup of temperature correction is shown in Figure 9.

We put the whole thermistor string into the GDJS-series high-low temperature test chamber over a range of −50 to 30 °C and measured the accuracy of single point of temperature sensor. To simulate the sea ice temperature measurement site, the temperature sensor of the thermistor string was immersed in antifreeze in a stainless steel container. A high-precision measuring instrument of temperature, which is a resistance temperature detector (RTD), with accuracy 0.001 °C fixed next to the temperature sensor was used to synchronously collect and record temperature data in the test chamber. The thermistor string and RTD were immersed in antifreeze. The antifreeze has a very low freezing point and can provide a stable temperature field for temperature measurement experiments. In order to make the temperature of the antifreeze more stable, we installed a motor-controlled impeller at constant speed at the bottom of the vessel. During the experiment, the interval of measured temperature is set as 5 °C from −50 °C to 30 °C. The baffle of the test chamber was closed for reducing the heat exchange between the test chamber and the outside.

**Figure 10:**
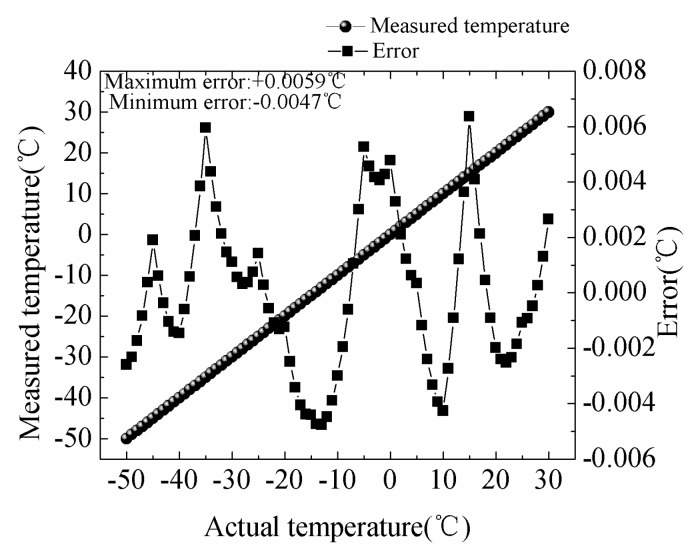
Measured temperature of the thermistor string.

The results of measured temperature of the thermistor string are shown in Figure 10. We observe that the discrepancy between actual temperatures and measured values remain minimal range. The maximum and minimum errors are 0.0059 °C at −35 °C and −0.0047 °C at −14 °C, respectively. The coefficient *a(T, I^2^)*, *b(T, I)*, *C(T)* can be calculated by directly measured temperature and the output of ADC circuit. The temperature measurement resolution of the thermistor string is 0.0001 °C and the sensitivity of the thermistor string is 0.1733 mV/ °C.

**Figure 11:**
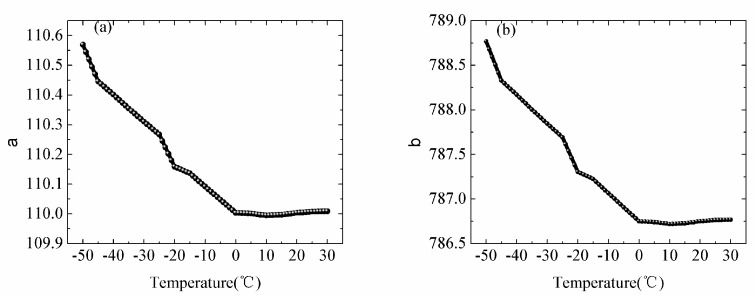
(**a**,**b**) show the temperature dependence of the coefficient a, b.

The coefficient *a(T, I^2^)* and *b(T, I)* decreases approximately in a parabolic manner with increase of temperature (Figure 11). The maximum and minimum *a(T, I^2^)* are 110.5689 at −50 °C and 109.994 at −10 °C, respectively. The maximum and minimum values of *b(T, I)* are 788.76 at −50 °C and 786.72 at −10 °C, respectively. The temperature dependence of coefficient *C(T)* can be ignored. The value of the coefficient *a(T, I^2^)* and *b(T, I)* at the nonmeasured temperatures could be predicted by linear interpolation.

## 5. Sea-Ice Thickness Discrimination Algorithm

The sea ice temperature profile observed by the multilayer sea ice temperature sensor includes a series of data that indicate the temperatures of air, snow, sea ice and ocean. Sea ice thickness can be calculated using the snow–ice interface and the ice–ocean interface [17]. Based on the clustering theory, we propose an automatic sea-ice thickness discrimination algorithm, which can effectively identify the two interfaces based on the sea ice temperature profile. Accordingly, accurate detection of the two interfaces can be abstracted to find two inflection points of the sea ice temperature profile. The data from the sea ice temperature profile can be viewed as a set of points to be processed. The sea ice temperature profile is divided into three subgroups, which can be described as follows. Suppose there are *n* points on the sea ice temperature profile.

(4) $$\begin{array}{l} T_{snow}=\mu_{snow}+e_{k},1\leq k\leq C_{1}\\ T_{ice}=\mu_{ice}+e_{k},C_{1}\leq k\leq C_{2}\\ T_{ocean}=\mu_{ocean}+e_{k},C_{2}\leq k\leq n \end{array}$$

where *T_snow_*, *T_ice_*, and *T_ocean_* are the temperatures of snow, ice, and ocean, respectively; *μ_snow_*, *μ_ice_*, and *μ_ocean_* are the expected values of temperature of snow, ice, and ocean, respectively; *e_k_* is the random error for every value; *C_1_* and *C_2_* are the locations of snow–ice and ice–ocean interfaces, respectively. Thus, the sea ice thickness can be calculated as follows.

(5) $$TH_{ice}=\left(C_{2}-C_{1}\right)•R$$

where *TH_ice_* is the thickness of sea ice; *R* represents the vertical spacing of adjacent temperature values.

The principle of the cluster theory is to take such an inflection point to minimize the sum of squares of the segment errors between the two subgroups which the inflection point is regarded as demarcation point, which target formula can be described as follows.

(6) $$C=arg\underset{1\leq k\leq n}{min}\left\{{\sum_{i=1}^{k-1}\left[T_{snow-ice}\left(i\right)-\mu_{snow-ice}\left(k\right)\right]}^{2}+{\sum_{i=k}^{n}\left[T_{ice-ocean}\left(i\right)-\mu_{ice-ocean}\left(k\right)\right]}^{2}\right\}$$

where *C* is the location of the interface; *T_snow-ice_* and *T_ice-ocean_* are the temperature value in a certain medium of snow or ice, and ice or ocean; *μ_snow-ice_* and *μ_ice-ocean_* and *μ_ocean_* are corresponding expected values and described as follows.

(7) $$\mu_{snow-ice}\left(k\right)=\frac{1}{k-1}•\sum_{i=1}^{k-1}T_{snow-ice}\left(i\right)$$

(8) $$\mu_{ice-ocean}\left(k\right)=\frac{1}{n-k+1}•\sum_{i=k}^{n}T_{ice-ocean}\left(i\right)$$

**Figure 12:**
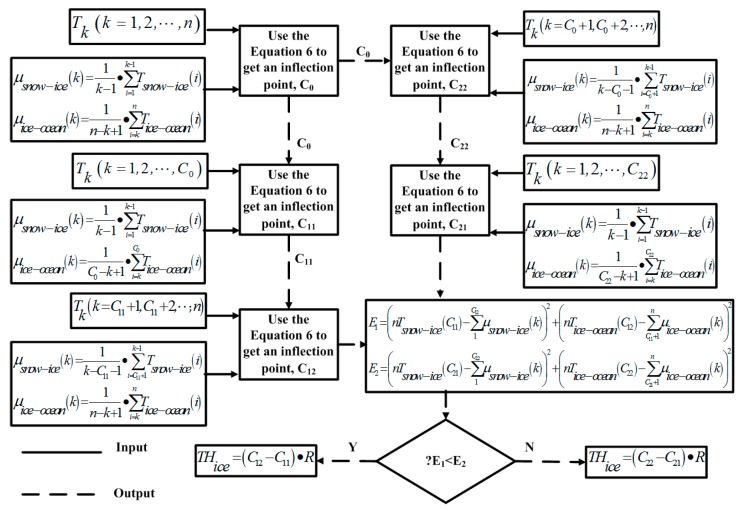
The flowchart of the sea ice thickness discrimination algorithm.

Figure 12 shows the flow chart of sea-ice thickness discrimination algorithm. Specifically, we use the Equation 6 to process data set of a sea ice temperature profile *T_k_* (k = 1, 2,…, *n*) to get an inflection point *C_0_*. Then a data set *T_k_* (k = 1, 2,…, *C_0_*) is processed using the same method to get an inflection point *C_11_*. For *T_k_* (k = *C_11_*+1, *C_11_*+2,…, *n*), we can obtain another inflection point *C_12_*. Similarly, we use the Equation 6 to process *T_k_* (k = *C_0_*+1, *C_0_*+2,…, n) to get an inflection point *C_22_*. Then a data set *T_k_* (k = 1, 2,…, *C_22_*) is processed using the same method to get an inflection point *C_21_*. So far we have got two sets of inflection points (*C_11_*, *C_12_*) and (*C_21_*, *C_22_*). *C_11_* and *C_21_* are potential snow–ice interfaces, and *C_12_* and *C_22_* are potential ice–ocean interfaces. As shown in Figure 12, if *E_1_ < E_2_*, *C_11_* and *C_12_* are two interfaces. Otherwise, *C_21_* and *C_22_* are two interfaces. The resolution of the calculated ice thickness is set 0.001 m.

## 6. Results of Field Experiment

### 6.1. Field Experiment in Wuliangsuhai, Yellow River 

**Figure 13:**
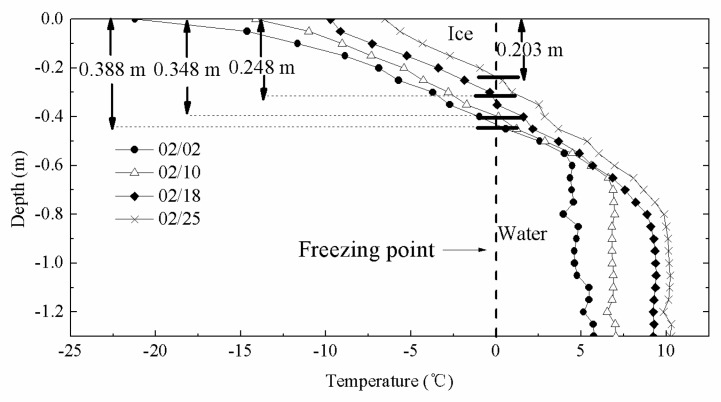
Temperature profiles of ice measured by Taiyuan University of Technology (TYUT) thermistor string.

Accuracy and stability of the multilayer sea ice temperature sensor was tested and evaluated by a field experiment in Wuliangsuhai, Yellow River (Figure 1b). We designed a 2.5 m long thermistor string assembled 50 PT1000. All PT1000 were soldered on FPCBs with interval of 0.05 m. FPCBs were nested in a 2.5 m long white polyolefin tube. Other thermal resistance instruments with accuracy of 0.01 °C were selected as comparison of thermistor string. The thermistor string and other thermal resistance instruments were installed in Wuliangsuhai, Yellow River on the frozen lake ice on 31 January 2018 and penetrated vertically through the air, ice, and lake water. The field experiment lasted from 1 February to 25 February. The initial ice thickness was 0.388 m and there was no snow cover on ice. Water depth of installation position was 1.4 m. Twenty-four PT1000 were exposed to the air, and 26 PT1000 were fixed in the ice. In particular, the lowest temperature sensor was installed 0.10 m from the bottom of the lake. The thermistor string can realize temperature data collection every 10 minutes. Other thermal resistance instruments were set to collect data every 5 minutes. In order to illustrate the results of the PT1000 thermistor string for observation of temperature profiles and determination of ice thickness, the four temperature profiles of lake ice at 8:00 were selected and analyzed in Figure 13. The vertical axis represents the distance of the temperature sensor from the air–ice surface.

As shown in Figure 13, according to the freezing point of freshwater ice (0 °C), the ice temperature profile and the water temperature profile can be clearly identified. Ice thickness can be calculated based on temperature profiles using sea-ice thickness discrimination algorithm. The ice thickness at this site continued to decrease until 25 February when the ice thickness reached its minimum value (0.203 m).

**Figure 14:**
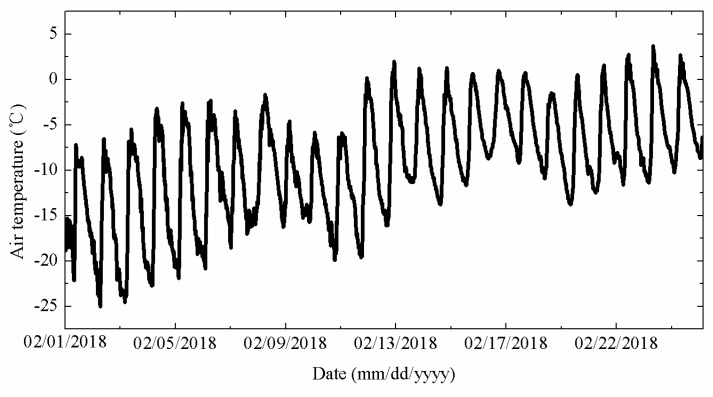
Air temperature measured by TYUT thermistor string from 1 February to 25 February.

The air temperature measured by the thermistor string from 1 February to 25 February is shown in Figure 14. The air temperature was presented a gradual upward trend which had strong fluctuations. The maximum (minimum) temperature was 3.68 °C (−25 °C), respectively. The average temperature was −9.2 °C.

**Figure 15:**
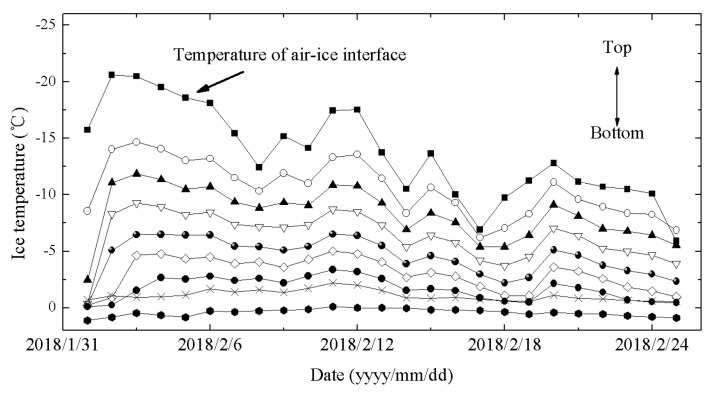
Ice temperature measured by TYUT thermistor string from 1 February to 25 February.

The ice temperature measured by the thermistor string from 1 February to 25 February is shown in Figure 15.

**Figure 16:**
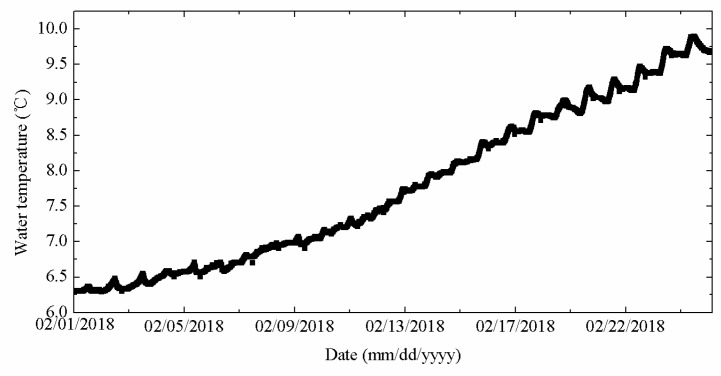
Water temperature measured by TYUT thermistor string from 1 February to 25 February.

In Figure 15, the nine temperature sensors from the top interface (air–ice interface) were selected. The temperatures of top interface (air–ice interface) were greatly affected by air temperature and the daily changes were relatively severe. For lower interface (ice–water interface), the heat mainly came from the lower water and bottom of the lake, so lower water temperature demonstrated high temperature. The water temperature measured by the thermistor string from 1 February to 25 February is shown in Figure 16. 

The water temperature was presented a gradual upward trend. The maximum (minimum) temperature was 9.9 °C (6.29 °C), respectively. The average temperature was 7.7 °C. An increase in water temperature may accelerate the melting of the ice, resulting in a gradual decrease in ice thickness from 1 February to 25 February.

**Figure 17:**
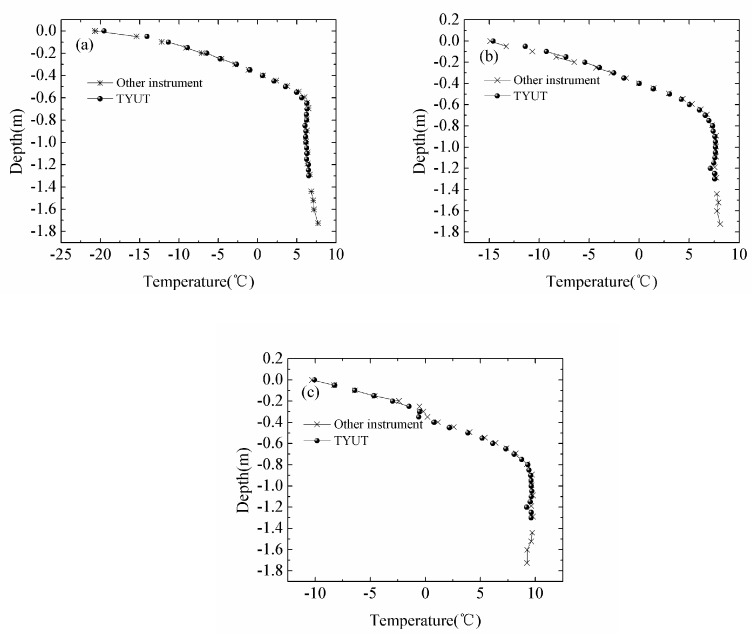
(**a**–**c**) are ice temperature curves of TYUT thermistor string and other instruments at 8:00 on 4 February, 13 February and 24 February, respectively.

Other instruments were installed near the thermistor string. However, the distance of temperature probes of the instruments differed from the thermistor string. The results of the comparison are shown in Figure 17. Figure 17 indicates that deviation between the results of temperature measurement by the thermistor string and other instruments is small. The main source of deviation may be the different conditions of installation location between two instruments although the installation location was close. And a slight inconsistency in the measurement layer may cause the deviation.

### 6.2. Field Experiment in the Second Reservoir of Fen River, Yellow River

**Figure 18:**
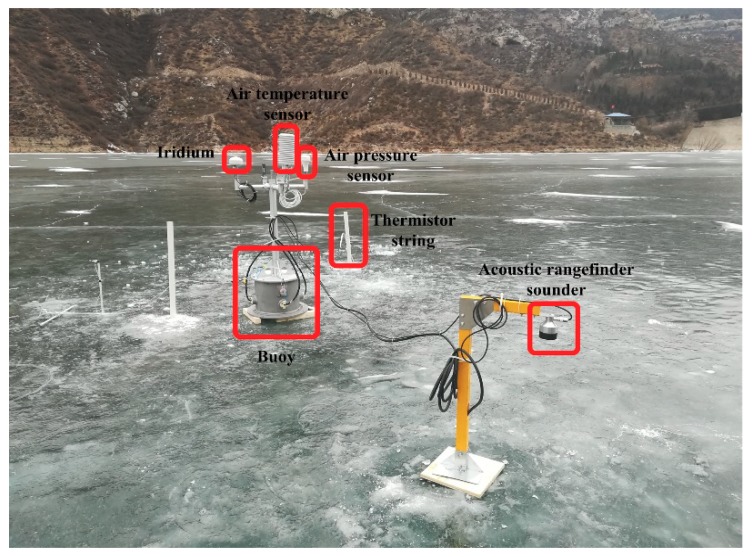
Deployment of the multilayer sea ice temperature sensor system in the second reservoir area of Fen River, Yellow River.

The multilayer sea ice temperature sensor was deployed in the second reservoir area of Fen River, Yellow River on 30 January 2018, as shown in Figure 18.

**Figure 19:**
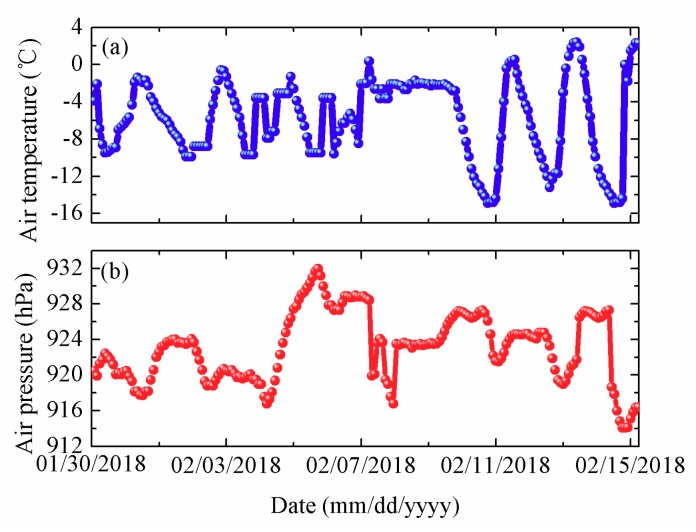
Air temperature (**a**) and air pressure (**b**) from 30 January to 15 February.

A 4.5 m long thermistor string was designed and assembled 150 PT1000. The sensor spacing of the thermistor string was 0.03 m. The sensor system integrated an air temperature sensor (HMP155A, Vaisala, Vantaa, Finland), an air pressure sensor (CS106/PTB110, Vaisala, Vantaa, Finland) and an Iridium module. The ice thickness was measured by drilled-hole once a day and two acoustic rangefinder sounders. The thermistor string and two acoustic rangefinder sounders were installed on the frozen lake ice. The field experiment lasted from 30 January to 15 February. The initial ice thickness was 0.29 m and there was no snow cover on ice. Water depth of installation position was 30.5 m. There were 25 temperature sensors exposed to the air, and others were fixed in the ice and water. The thermistor string was in charge of realizing temperature data collection every two hours. The air temperature and air pressure from 30 January to 15 February are shown in Figure 19.

As shown in Figure 19a, the air temperature was presented a trend of fluctuations. From the 30 January to 7 February, the amplitude of the air temperature change was small, and then until 15 February, the amplitude became larger. The maximum (minimum) air temperature was 2.40 °C (−14.9 °C), respectively. The average temperature was −5.68 °C. In Figure 19b, the maximum (minimum) air pressure was 931.9 hPa (914.1 hPa), respectively. And the average air pressure was 923.1 hPa.

**Figure 20:**
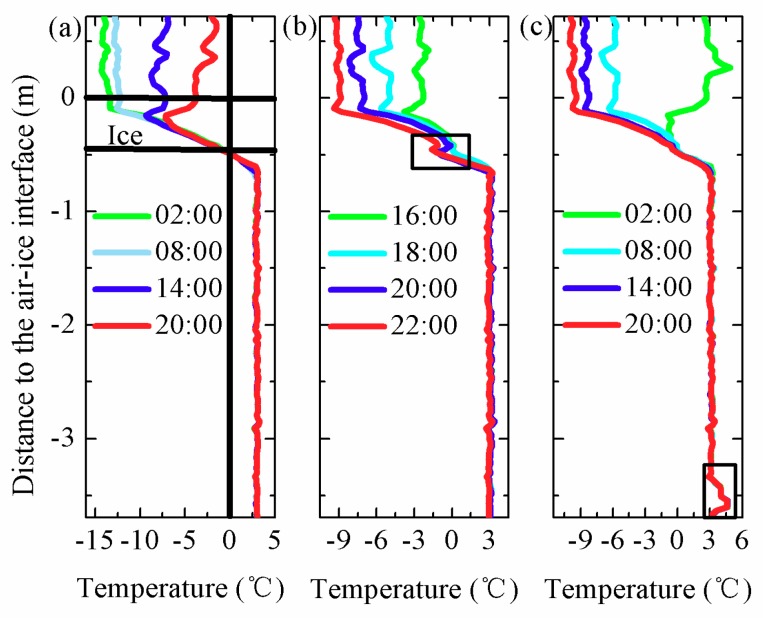
(**a**) Temperature profiles at 02:00, 08:00, 14:00 and 20:00 on 11 February. (**b**) Temperature profiles at 16:00, 18:00, 20:00 and 22:00 on 13 February. (**c**) Temperature profiles at 02:00, 08:00, 14:00 and 20:00 on 14 February. Black boxes indicate abnormal temperature values.

Typical temperature profiles are shown in Figure 20. Figure 20a indicates temperature profiles measured by the thermistor string at 02:00, 08:00, 14:00 and 20:00 on 11 February. As mentioned above, there were 25 temperature sensors exposed to the air, thus, the position of the 25th sensor can be regarded as the air–ice interface. The intersection of the temperature profile and 0 °C (freezing point) can be considered the bottom of the ice. Therefore, the ice thickness can be identified based on the temperature profiles. On 13 February 2018, the dam officials lowered reservoir levels and tiny gaps appeared between ice and water. As shown in Figure 20b, the temperature values flipping occurred near the intersection between temperature profiles at 16:00, 18:00, 20:00 and 22:00 on 13 February and 0 °C. On 13 February 2018, the dam reduced flow and stored water. At this time, a small amount of warm water upstream was trapped near the dam. In Figure 20c, abnormal local high water temperature was observed at the bottom of the temperature profiles at 02:00, 08:00, 14:00 and 20:00 on 14 February. This phenomenon is consistent with the actual operation of the reservoir.

**Figure 21:**
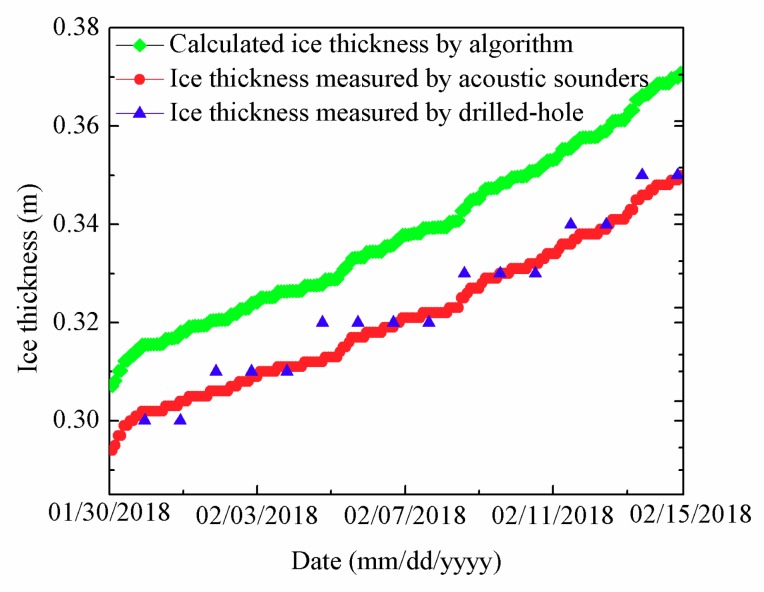
Calculated ice thickness by algorithm (**green lines**), ice thickness measured by acoustic sounders (**red lines**), and ice thickness measured by drilled-hole (**blue triangles**) from 30 January to 15 February.

Calculated ice thickness by algorithm (green lines), ice thickness measured by acoustic sounders (red lines), and ice thickness measured by drilled-hole (blue triangles) from 30 January to 15 February are shown in Figure 21. As can be easily seen, ice thickness measured by acoustic sounders was consistent with ice thickness measured by drilled-hole. The maximum (minimum) error of ice thickness between acoustic sounders data and drilled-hole data was 0.004 m (−0.008 m), respectively. The average error of ice thickness was −0.013 m. The calculated ice thickness by algorithm and ice thickness measured by acoustic sounders had the same trend. The maximum (minimum) ice thickness measured by acoustic sounders was 0.35 m (0.294 m), respectively. The average ice thickness measured by acoustic sounders was 0.32 m. The maximum (minimum) ice thickness calculated by algorithm was 0.37 m (0.30 m), respectively. The average ice thickness calculated by algorithm was 0.338 m. The maximum (minimum) error of ice thickness between acoustic sounders data and algorithm data was 0.021 m (0.013 m), respectively. The average error of ice thickness between acoustic sounders data and algorithm data was 0.017 m.

## 7. Conclusions

A multilayer sea ice temperature sensor has been designed in order to realize high-precision temperature measurement at the operating range of −50 °C to 30 °C. The sensor system composition, structure of the thermistor string, and work mode of the sensor system are depicted in detail. The multilayer sea ice temperature sensor includes a thermistor string for measuring sea ice temperature profile and a buoy, which is easy to be deployed and installed. The thermistor string is composed of four layers of a heat-shrink soft tube, a hot-melt adhesive, an expoxy resin, and a FPCB. We comprehensively evaluated low-temperature performances of this sensor system by experiments simulating low temperature in the polar regions. The experimental setups of the sensor system are built, which involves temperature dependence of the constant current source, ADC circuit and amplifier. The results of performance evaluation of sensor system indicate that the sensor system has excellent stability over −50 to 30 °C. Compared to traditional sea ice observation instruments, the sensor system has higher temperature measurement accuracy. And a correction algorithm based on the experiment model is developed to correct the biases to achieve temperature measurement error from −0.0047 °C to 0.0059 °C. Additionally, an automatic sea ice thickness discrimination algorithm is proposed. Field experiments were completed and the sensor system can be proved high precision and high stability in observation of ice temperature profile in Wuliangsuhai, Yellow River. During the field experiment in the second reservoir of Fen River, Yellow River, anomalies in temperature profiles were observed by the sensor system, which may be caused by releasing and storing water of dam. And successful observation of this phenomenon indicates that the sensor system has high sensitivity of measuring the multilayer ice temperature. Therefore, the multilayer sea ice temperature sensor is considered suitable for application of the multilayer temperature measurement of sea ice in polar regions.

More design optimization can be realized to create a sensor system with better performance. In the future work, an integrated structure of the buoy will be designed and implemented, which the thermistor string will be integrated on the buoy, and the external cable can be removed. The length of the thermistor string can be further enlarged. For example, a 50 m long thermistor string can be designed to be in charge of measuring the ice cover temperature field in the Arctic Ocean and Antarctica. More sensors with different parameters, such as conductivity-temperature-depth (CTD) sensor, pressure sensor chain, etc, can be assembled into the sensor system to obtain a lot of data of sea ice and ocean. The sensor systems can be widely deployed and installed in the Arctic Ocean and Antarctica to improve the accuracy of temperature correction algorithm and the applicability of sea-ice thickness discrimination algorithm through in situ observation. 

The ice volume is calculated from the ice thickness and the sea ice concentration. Comparison of Satellite measurements (CryoSat-2) with some in-situ data sets reveals differences of less than 0.1 m in thickness [18]. However, the error of ice thickness between calculation by the discrimination algorithm and actual ice thickness remains in a small range (0.013 to 0.017 m). Thus, the inverted ice thickness by our algorithm may have an advantage in the estimation of sea ice volume. At present, there is not a lot of the continuous in-situ ice thickness data, or the spatially more representative ice thickness data. The application of ice thickness discrimination algorithm is limited. In future work, the large deployment of sea ice temperature sensors or other sea ice temperature observation systems in the Arctic Ocean and Antarctica can improve the calculation accuracy of ice volume and reveal the mechanism of sea ice changes in the polar regions.

## Figures and Tables

**Table 1:** States of the art in sea ice temperature sensor.

Sensor	Temperature Measurement Accuracy ( °C)	Length (m)	Sensor Spacing (m)	Structure
Ice mass balance buoy (IMB)	0.1	4.5	0.10	Solid state
Sea ice mass balance buoy (SIMB)	0.1	4.8	0.02	Flexible

**Table 2:** Size parameters of the four layers.

Parameter	Value	Unit
D_1_	0.03	m
D_2_	0.025	m
D_3_	0.022	m
D_4_	0.01	m
D_5_	7.5	mm
D_6_	6.0	mm
D_7_	4.5	mm
D_8_	2.5	mm

## References

[1] Screen J.A., Simmonds I. (2010). The central role of diminishing sea ice in recent Arctic temperature amplification. Nature.

[2] Kwok R., Rothrock D.A. (2009). Decline in Arctic sea ice thickness from submarine and ICESat records: 1958–2008. Geophys. Res. Lett..

[3] Rothrock D.A., Percival D.B., Wensnahan M. (2008). The decline in arctic sea-ice thickness: Separating the spatial, annual, and interannual variability in a quarter century of submarine data. J. Geophys. Res. Oceans.

[4] Wang C.C., Hou Z.Y., You J.C. (2018). A high-precision CMOS temperature sensor with thermistor linear calibration in the (−5 °C, 120 °C) temperature range. Sensors.

[5] Jamieson J. (1991). A platinum resistance thermometer. Electr. Educ..

[6] Arenas O., Alam É.A., Thevenot A., Cordier Y., Jaouad A., Aimez V., Maher H., Arès R., Boone F. (2014). Integration of micro resistance thermometer detectors in AlGaN/GaN devices. IEEE J. Electron Devices Soc..

[7] Perovich D.K., Grenfell T.C., Light B. (2009). Transpolar observations of the morphological properties of Arctic sea ice. J. Geophys. Res..

[8] Perovich D.K., Elder B. (2002). Estimates of ocean heat flux at SHEBA. Geophys. Res. Lett..

[9] Lei R.B., Li N., Heil P. (2014). Multiyear sea ice thermal regimes and oceanic heat flux derived from an ice mass balance buoy in the Arctic Ocean. J. Geophys. Res. Oceans.

[10] Lei R.B., Cheng B., Vihma T. (2018). Seasonal and Interannual Variations of Sea Ice Mass Balance From the Central Arctic to the Greenland Sea. J. Geophys. Res. Oceans.

[11] Richter-Menge J.A., Perovich D.K., Elder B. (2006). Ice mass balance buoys: A tool for measuring and attributing changes in the thickness of the Arctic sea ice cover. Ann. Glaciol..

[12] Jackson K., Wilkinson J., Maksym T. (2013). A novel and low cost sea ice mass balance buoy. J. Atmos. Oceanic Technol..

[13] McPhee M.G., Untersteiner N. (1982). Using sea ice to measure heat flux in the ocean. J. Geophys. Res. Oceans.

[14] Cheng B., Zhang Z.H., Vihma T. (2008). Model experiments on snow and ice thermodynamics in the Arctic Ocean with CHINARE 2003 data. J. Geophys. Res..

[15] Hoppmann M., Nicolaus M., Hunkeler P.A. (2015). Seasonal evolution of an ice-shelf influenced fast-ice regime, derived from an autonomous thermistor chain. J. Geophys. Res. Oceans.

[16] Provost C., Sennéchael N., Miguet J. (2017). Observations of flooding and snow-ice formation in a thinner Arctic sea ice regime during the N-ICE2015 campaign: Influence of basal ice melt and storms. J. Geophys. Res. Oceans.

[17] Zuo G.Y., Dou Y.K., Lei R.B. (2018). Discrimination Algorithm and Procedure of Snow Depth and Sea Ice Thickness Determination Using Measurements of the Vertical Ice Temperature Profile by the Ice-tethered Buoys. Sensors.

[18] Laxon S.W., Giles K.A., Ridout A.L. (2013). CryoSat-2 estimates of Arctic sea ice thickness and volume. Geophys. Res. Lett..

